# Association between heated tobacco product use and worsening asthma symptoms: findings from a nationwide internet survey in Japan, 2023

**DOI:** 10.1265/ehpm.25-00197

**Published:** 2025-09-30

**Authors:** Shingo Noguchi, Tomohiro Ishimaru, Kazuhiro Yatera, Yoshihisa Fujino, Takahiro Tabuchi

**Affiliations:** 1 Department of Respiratory Medicine, University of Occupational and Environmental Health, Japan; 2 Department of Respiratory Medicine, Tobata General Hospital; 3 Department of Medical Humanities, School of Medicine, University of Occupational and Environmental Health, Japan; 4 Department of Environmental Epidemiology, Institute of Industrial Ecological Sciences, University of Occupational and Environmental Health, Japan; 5 Division of Epidemiology, School of Public Health, Tohoku University Graduate School of Medicine

**Keywords:** Adults, Asthma, Allergic disease, Cigarette, Heated tobacco products

## Abstract

**Background:**

Heated tobacco products (HTPs) are widely used in Japan, following cigarettes, but their health effects remain unclear. HTPs are often considered a less harmful alternative to cigarettes and are commonly used by adults with asthma, even though smoking is one of the most obvious and treatable factors in asthma. We aimed to elucidate the association between HTP use and asthma symptoms in adults with asthma.

**Methods:**

A total of 3,787 individuals with asthma were extracted from the data in the Japan COVID-19 and Society Internet Survey 2023, an ongoing longitudinal internet-based cohort study conducted by a nationwide internet research company in Japan. They were categorized into three groups (never, past, and current smokers) based on cigarette use. The association between HTP use and worsening of asthma symptoms within the previous 2 months in each group was analyzed using univariate and multivariate logistic regression analyses. Both exposure and outcomes were assessed by self-reporting.

**Results:**

Among the participants, 2,470 (65.2%) were never smokers, 845 (22.3%) were past smokers, and 472 (12.5%) were current smokers. Overall, the proportion of HTP users was 429 (11.3%), and worsened asthma symptoms were observed in 400 (10.6%) individuals. The total proportion of HTP users and worsened asthma symptoms was 70 (2.8%) and 259 (10.5%) among never smokers, 180 (21.3%) and 72 (8.5%) among past smokers, and 179 (37.9%) and 69 (14.6%) among current smokers. After adjusting for confounders, the odds ratio (OR) was 3.25 (95% confidence interval [CI] 1.86–5.68, p < 0.001), 1.47 (95% CI 0.93–2.34, p = 0.1), and 2.23 (95% CI 1.46–3.43, p < 0.001) for never, past, and current cigarette smokers with HTP use, respectively, where never smokers without HTP use were set as the standard.

**Conclusion:**

The use of HTPs, not only cigarette smoking, was associated with worsening of asthma symptoms in adults with asthma. Therefore, people need to understand the harmful effects of HTPs on asthma symptoms.

## Background

Asthma is a common chronic disease worldwide, with a global prevalence of approximately 6–7% in adults, although the proportions differ by region [1]. In Japan, the prevalence of adult asthma is reportedly 6–10%, and analysis using Japanese health insurance claims data demonstrated a steady increase in asthma prevalence since 1999; it increased by approximately 1.5-fold between 2011 and 2019 [2, 3].

Asthma is characterized by chronic airway inflammation and symptoms such as cough, wheezing, dyspnea, chest tightness caused by airflow limitation and airway hyperresponsiveness [4, 5]. Management in clinical practice aims to control the symptoms and prevent asthma exacerbation, annual decline in lung function, and asthma-related death [5]. Although asthma-related mortality has declined in recent years due to advances in treatment options, it has plateaued at around 1,000 deaths annually in Japan [5, 6]. Poor symptom control and asthma exacerbations are associated with an increased risk of severe outcomes [7]; therefore, it is essential to maintain good daily asthma control.

Cigarette smoking is associated with poor asthma control, increased hospitalization and mortality, and decreased quality of life in individuals with asthma [8–13]; therefore, it is one of the most obvious and treatable factors in asthma management. The prevalence of cigarette smoking differs widely across countries, but high rates of cigarette use in patients with asthma are prevalent worldwide [9, 14–16], with a high proportion (5.8–18.9%) in Japan [10, 17–19], despite the importance of quitting cigarette smoking.

Heated tobacco products (HTPs) were first launched in Japan and Italy in 2014, and have rapidly expanded to over 60 countries worldwide [20]. Globally, the prevalence of HTP use is increasing but remains relatively low overall. A meta-analysis estimated the pooled global prevalence of current HTP use to be 1.53% between 2015 and 2022, with the highest rate observed in Japan (10.9%) and South Korea (4.7%) [20]. In Japan, HTPs are the second most common products, following cigarettes. Japanese cigarette manufacturers promote HTPs as a means to quit smoking, despite evidence suggesting that their use is strongly associated with relapse into, or resumption of, regular cigarette smoking [21].

The Japan COVID-19 and Society Internet Survey (JACSIS) study is a large-scale, nationwide, internet-based cohort study designed to investigate tobacco-related behaviors, health status, sociodemographic factors, etc. It collects data on cigarette and HTP use in Japan and continues to monitor trends in tobacco product use. The prevalence of HTP use in Japan increased by approximately 50-fold, from 0.2% in 2015 to 10.9% in 2020, and has since remained persistently high at around 11–12%, according to the JACSIS study [22, 23]. HTPs are often considered a less harmful alternative to cigarettes [24], but the health effects of short- and long-term use of HTPs remain unclear. Additionally, HTPs are strongly associated with the resumption of regular cigarette smoking, and users often tend to be dual users of both products [21]. We previously reported that HTP use was high in adults with asthma and chronic obstructive pulmonary disease (COPD), especially in current cigarette smokers [25]; however, the influence of HTPs in adults with chronic respiratory and allergic diseases such as asthma is unclear.

In the present study, we aimed to clarify the association between HTP use and asthma symptoms in adults with asthma, a common respiratory disease in all age groups, using a large-scale nationwide internet survey.

## Methods

### Data source

Data from JACSIS 2023 were used in this study. The JACSIS survey is an ongoing longitudinal internet-based cohort study conducted by a nationwide internet research company in Japan (Rakuten Insight Corporation, Tokyo, Japan) [26], and the present analysis was based on cross-sectional data. Briefly, participants were recruited through simple random sampling using a computer algorithm from the Rakuten Insight survey panel, which comprises approximately 2.2 million panelists. Sampling was stratified by age, sex, and residential prefecture to reflect the demographic composition of the Japanese population [27]. Recruitment continued until the target number of participants was reached. This JACSIS 2023 survey was performed from September 25 to November 17, 2023. Initially, invitation e-mails for study participation were sent to 46,840 participants who had been pooled in the follow-up period between 2015 to 2023, and 26,872 participants were enrolled (response rate, 57.4%). Additionally, 6,128 participants aged 16–79 years were recruited from the panel members [28]. Ultimately, the total number of participants was 33,000. This study was approved by the Research Ethics Committee of the Osaka International Cancer Institute (No. 20084-9, approved on February 16, 2023) and was conducted in accordance with the principles of the Declaration of Helsinki. All participants provided web-based written informed consent obtained via the internet at the time of enrolment.

### Inclusion and exclusion criteria

Data from the first 33,000 respondents from the 2023 survey were included in the initial dataset. Respondents without asthma and those with inconsistent responses were excluded. Invalid responses were defined to ensure the survey’s validity and included: failing to select the correct answer to the question, “Please select the second lowest of five options.”; reporting the use of all drugs in response to, “Do you currently use any of eight drugs?”; selecting all items for the question, “Do you currently have any chronic diseases?”; reporting more than 15 people living together; and providing answers in under 15 min.

### Definition of asthma and worsening asthma symptoms

Participants were considered to have asthma if they reported having it in the present or past, regardless of whether they had been hospitalized or received treatment for it, in response to the question, “Do you currently have any chronic diseases?” Participants were considered to have experienced asthma worsening if they answered “yes” to the question, “Have you had worsening asthma symptoms in the last 2 months?” This was applicable to those who had indicated that they had asthma.

### Definition of cigarette and HTP use

Participants were classified as “current cigarette or HTP smokers” if they indicated that they often or sometimes used any tobacco products in response to the question, “Do you currently use each tobacco product?” Those identified as “former cigarette or HTP smokers” had used these products regularly in the past but were not currently using them. Participants who had never smoked or had only smoked one cigarette without ever using tobacco products regularly were categorized as “never smokers.” “Former HTP smokers” and “never HTP smokers” were classified as “no HTP use.” Tobacco products were categorized as follows: cigarette use (paper-wrapped cigarettes) and HTP use (Ploom Tech, Ploom X, IQOS, Glo, and lil HYBRID).

### Other variables

The variables included age groups (16–29, 30–39, 40–49, 50–59, 60–69, and 70–83 years), sex, educational attainment (junior high or high school, vocational school or college, and university or graduate school), annual household income (<2,000,000 yen, 2,000,000–3,999,999 yen, 4,000,000–6,999,999 yen, and ≥7,000,000 yen, unknown), alcohol intake (never, occasionally, and regular), and body mass index (BMI) (<18.5, 18.5–24.9, 25.0–29.9, ≥30.0 kg/m^2^).

### Statistical analyses

Univariate and multivariate logistic regression analyses were performed to investigate the association between HTP use and worsening asthma symptoms. The participants were classified into six groups according to their smoking history and HTP use: (1) never cigarette smokers without HTP use (reference group); (2) never cigarette smokers with HTP use; (3) past cigarette smokers without HTP use; (4) past cigarette smokers with HTP use; (5) current cigarette smokers without HTP use; and (6) current cigarette smokers with HTP use. Multivariate logistic regression was used to adjust for potential confounders, including age, sex, educational attainment, annual household income, alcohol intake, and BMI. Additionally, the odds ratios (ORs) and 95% confidence intervals (CIs) were calculated for these confounding variables to assess their independent associations with worsening asthma symptoms. All statistical analyses were conducted using Stata/SE 16.1 (StataCorp, College Station, TX, USA), with statistical significance set at p < 0.05.

## Results

**Fig. 1:**
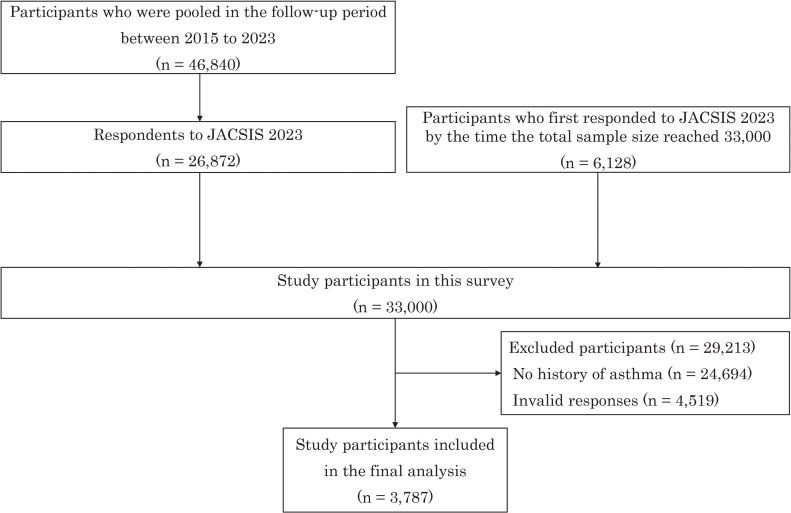
Flow chart of the study participants.

Among the 33,000 participants, 24,694 with no history of asthma and 4,519 with invalid responses were excluded, and 3,787 participants were eventually included in this study (Fig. 1). The demographic characteristics of the patients with asthma are presented in Table 1. Among these 3,787 participants, those aged 16–29 accounted for the largest proportion (n = 1,024, 27.0%), and the number of men was 1,904 (50.3%). The rates of “never,” “past,” and “current” cigarette smokers were 2,470 (65.2%), 845 (22.3%), and 472 (12.5%), respectively. The total proportion of HTP users was 429 (11.3%), with 70 (2.8%) among never smokers, 180 (21.3%) among past smokers, and 179 (37.9%) among current smokers. The proportion of participants with worsening asthma symptoms was 400 (10.6%), and the total proportion of individuals with worsened asthma symptoms was 259 (10.5%) among never smokers, 72 (8.5%) among past smokers, and 69 (14.6%) among current smokers.

**Table 1:** Demographic characteristics of participants

	**Never cigarette smokers**		**Past cigarette smokers**		**Current cigarette smokers**		**Total**
		
	**HTPs (−)**	**HTPs (+)**	**HTPs (−)**	**HTPs (+)**	**HTPs (−)**	**HTPs (+)**	
	**n = 2,400**	**n = 70**	**n = 665**	**n = 180**	**n = 293**	**n = 179**	**N = 3,787**
Age (years)							
16–29	803 (33.5)	27 (38.6)	59 (8.9)	49 (27.2)	36 (12.3)	50 (27.9)	1,024 (27.0)
30–39	450 (18.8)	19 (27.1)	57 (8.6)	28 (15.6)	41 (14.0)	42 (23.5)	637 (16.8)
40–49	398 (16.6)	11 (15.7)	127 (19.1)	52 (28.9)	62 (21.2)	34 (19.0)	684 (18.1)
50–59	294 (12.3)	8 (11.4)	134 (20.2)	29 (16.1)	78 (26.6)	30 (16.8)	573 (15.1)
60–69	248 (10.3)	3 (4.3)	158 (23.8)	18 (10.0)	49 (16.7)	18 (10.1)	494 (13.0)
70–83	207 (8.6)	2 (2.9)	130 (19.5)	4 (2.2)	27 (9.2)	5 (2.8)	375 (9.9)
Sex							
Females	1,417 (59.0)	18 (25.7)	237 (35.6)	64 (35.6)	101 (34.5)	46 (25.7)	1,883 (49.7)
Males	983 (41.0)	52 (74.3)	428 (64.4)	116 (64.4)	192 (65.5)	133 (74.3)	1,904 (50.3)
Educational attainment							
Junior high or high school	552 (23.0)	21 (30.0)	232 (34.9)	62 (34.4)	99 (33.8)	43 (24.0)	1,009 (26.6)
Vocational school or college	543 (22.6)	12 (17.1)	124 (18.6)	46 (25.6)	59 (20.1)	30 (16.8)	814 (21.5)
University or graduate school	1,305 (54.4)	37 (52.9)	309 (46.5)	72 (40.0)	135 (46.1)	106 (59.2)	1,964 (51.9)
Annual household income (yen)							
<2,000,000	186 (7.8)	5 (7.1)	65 (9.8)	12 (6.7)	39 (13.3)	12 (6.7)	319 (8.4)
2,000,000–3,999,999	463 (19.3)	18 (25.7)	139 (20.9)	28 (15.6)	58 (19.8)	38 (21.2)	744 (19.6)
4,000,000–6,999,999	612 (25.5)	18 (25.7)	177 (26.6)	49 (27.2)	87 (29.7)	48 (26.8)	991 (26.2)
≥7,000,000	627 (26.1)	20 (28.6)	172 (25.9)	59 (32.8)	62 (21.2)	52 (29.1)	992 (26.2)
Unknown	512 (21.3)	9 (12.9)	112 (16.8)	32 (17.8)	47 (16.0)	29 (16.2)	741 (19.6)
Alcohol intake							
Never	1,473 (61.4)	52 (74.3)	256 (38.5)	96 (53.3)	120 (41.0)	64 (35.8)	2,061 (54.4)
Occasionally	695 (29.0)	11 (15.7)	209 (31.4)	53 (29.4)	91 (31.1)	62 (34.6)	1,121 (29.6)
Regular	232 (9.7)	7 (10.0)	200 (30.1)	31 (17.2)	82 (28.0)	53 (29.6)	605 (16.0)
Body mass index (kg/m^2^)							
<18.5	1,607 (67.0)	44 (62.9)	440 (66.2)	117 (65.0)	166 (56.7)	111 (62.0)	477 (12.6)
18.5–24.9	356 (14.8)	7 (10.0)	40 (6.0)	22 (12.2)	36 (12.3)	16 (8.9)	2,485 (65.6)
25.0–29.9	329 (13.7)	12 (17.1)	144 (21.7)	31 (17.2)	68 (23.2)	32 (17.9)	616 (16.3)
≥30.0	108 (4.5)	7 (10.0)	41 (6.2)	10 (5.6)	23 (7.8)	20 (11.2)	209 (5.5)
Type of HTP							
Ploom TECH		23 (32.9)		34 (18.9)		56 (31.3)	113 (3.0)
Ploom X		18 (25.7)		38 (21.1)		63 (35.2)	119 (3.1)
IQOS		37 (52.9)		86 (47.8)		103 (57.5)	226 (6.0)
Glo		17 (24.3)		56 (31.1)		67 (37.4)	140 (3.7)
lil HYBRID		14 (20.0)		23 (12.8)		30 (16.8)	67 (1.8)

HTPs, heated tobacco products

**Fig. 2:**
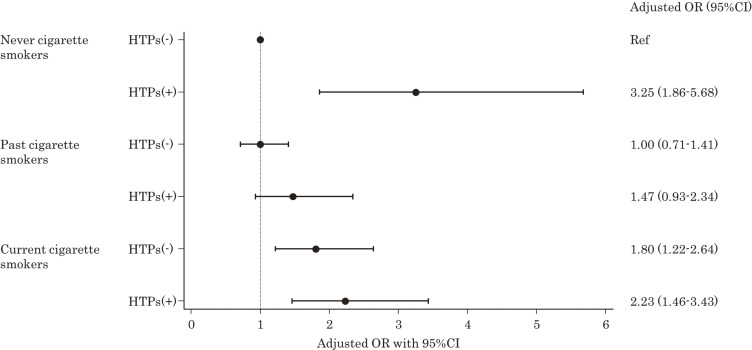
Adjusted ORs (95% CI) for worsening asthma symptoms by HTP use and cigarette smoking status The reference group were never-cigarette smokers without a history of HTP use. Dots represent the estimated adjusted ORs, and horizontal lines indicate the corresponding 95% CIs. The horizontal axis represents the adjusted ORs. ORs, odds ratios; CI, confidence interval

Table 2 presents the univariate and multivariate adjusted OR for the association between HTP use and worsening asthma symptoms. After adjusting for age, sex, educational attainment, annual household income, alcohol intake, and BMI, the adjusted ORs (95% CIs) for worsening asthma symptoms were 3.25 (1.86–5.68) among participants using HTPs compared to those not using HTPs among never cigarette smokers. The adjusted ORs were 1.00 (0.71–1.41) for participants without HTP use and 1.47 (0.93–2.34) for participants with HTP use among past cigarette smokers. Furthermore, the adjusted ORs were 1.80 (1.22–2.64) for participants without HTP use and 2.23 (1.46–3.43) for those with HTP use among current cigarette smokers (Fig. 2). The adjusted ORs for worsening asthma symptoms in older individuals were significantly lower than those of individuals aged 16–29 years; the adjusted ORs (95% CIs) were 0.73 (0.54–0.99), 0.71 (0.52–0.97), 0.44 (0.30–0.64), 0.47 (0.32–0.71), and 0.41 (0.25–0.67) in persons with asthma aged “30–39,” “40–49,” “50–59,” “60–69,” and “70–83,” years, respectively, compared with those aged “16–29” years.

**Table 2:** Association between HTP use and worsening asthma symptoms.

	**Total**	**Worsening asthma symptoms**		**Univariable**			Multivariable*		
			
	**N**	**n**	**%**	**OR**	**(95% CI)**	***P* value**	**OR**	**(95% CI)**	***P* value**
Smoking status									
Never cigarette smokers									
HTPs (−)	2,400	240	10.0	1.00	-	-	1.00	-	-
HTPs (+)	70	19	27.1	**3.35**	**(1.95–5.77)**	**<0.001**	**3.25**	**(1.86–5.68)**	**<0.001**
Past cigarette smokers									
HTPs (−)	665	48	7.2	**0.70**	**(0.51–0.97)**	**0.03**	1.00	(0.71–1.41)	0.984
HTPs (+)	180	24	13.3	1.39	(0.88–2.17)	0.156	1.47	(0.93–2.34)	0.1
Current cigarette smokers									
HTPs (−)	293	38	13.0	1.34	(0.93–1.93)	0.116	**1.80**	**(1.22–2.64)**	**0.003**
HTPs (+)	179	31	17.3	**1.89**	**(1.25–2.84)**	**0.002**	**2.23**	**(1.46–3.43)**	**<0.001**
Age (years)									
16–29	1,024	151	14.7	1.00	-	-	1.00	-	
30–39	637	76	11.9	0.78	(0.58–1.05)	0.105	**0.73**	**(0.54–0.99)**	**0.042**
40–49	684	75	11.0	**0.71**	**(0.53–0.96)**	**0.024**	**0.71**	**(0.52–0.97)**	**0.033**
50–59	573	41	7.2	**0.45**	**(0.31–0.64)**	**<0.001**	**0.44**	**(0.30–0.64)**	**<0.001**
60–69	494	35	7.1	**0.44**	**(0.30–0.65)**	**<0.001**	**0.47**	**(0.32–0.71)**	**<0.001**
70–83	375	22	5.9	**0.36**	**(0.23–0.57)**	**<0.001**	**0.41**	**(0.25–0.67)**	**<0.001**
Sex									
Females	1,883	201	10.7	1.00	-	-	1.00	-	
Males	1,904	199	10.5	0.98	(0.79–1.20)	0.824	0.87	(0.69–1.09)	0.229
Educational attainment									
Junior high or high school	1,009	98	9.7	1.00	-	-	1.00	-	
Vocational school or college	814	87	10.7	1.11	(0.82–1.51)	0.493	1.14	(0.83–1.56)	0.421
University or graduate school	1,964	215	10.9	1.14	(0.89–1.47)	0.299	1.08	(0.82–1.41)	0.599
Annual household income (yen)									
<2,000,000	319	36	11.3	1.00	-	-	1.00	-	
2,000,000–3,999,999	744	69	9.3	0.80	(0.53–1.23)	0.315	0.81	(0.52–1.25)	0.336
4,000,000–6,999,999	991	115	11.6	1.03	(0.69–1.54)	0.877	0.98	(0.65–1.47)	0.912
≥7,000,000	992	113	11.4	1.01	(0.68–1.51)	0.959	1.00	(0.66–1.52)	0.999
Unknown	741	67	9.0	0.78	(0.51–1.20)	0.259	0.74	(0.48–1.15)	0.183
Alcohol intake									
Never	2,061	249	12.1	1.00	-	-	1.00	-	
Occasionally	1,121	105	9.4	**0.75**	**(0.59–0.96)**	**0.02**	**0.74**	**(0.58–0.95)**	**0.019**
Regular	605	46	7.6	**0.60**	**(0.43–0.83)**	**0.002**	**0.67**	**(0.47–0.95)**	**0.025**
Body mass index (kg/m^2^)									
<18.5	477	51	10.7	0.98	(0.72–1.35)	0.911	0.86	(0.62–1.19)	0.362
18.5–24.9	2,485	270	10.9	1.00	-	-	1.00	-	
25.0–29.9	616	61	9.9	0.90	(0.67–1.21)	0.489	1.00	(0.74–1.36)	0.981
≥30.0	209	18	8.6	0.77	(0.47–1.27)	0.313	0.77	(0.46–1.28)	0.315

HTPs, heated tobacco products; OR, odds ratio; CI, confidence interval.

*Adjusted for smoking status, age, sex, educational attainment, annual household income, alcohol intake, and BMI.

## Discussion

In the present study, we investigated the association between HTP use and asthma symptoms in adults with asthma and found that HTP use, in addition to cigarette smoking, is associated with worsening asthma symptoms.

Regarding the association between HTP use and asthma, some studies targeting Korean adolescents have indicated that the ever-use of HTPs increases the morbidity risk of patients with current asthma [29, 30]. Similarly, Japanese reports have indicated that passive exposure to HTP aerosols is associated with increased asthma attacks and persistent cough in adults with asthma [31, 32]. However, evidence regarding the association between HTP use and asthma symptoms is limited, and there is no evidence of the direct influence of HTP use on asthma symptoms. Regarding the influence of HTP use on airflow restriction, Odani et al. showed that airway obstruction was higher in HTP users than in individuals who had never used HTPs or cigarettes (adjusted prevalence ratio 2.32, 95%CI 1.54–3.49) among adults with cancer [33]. Additionally, Harada et al. also demonstrated that HTP use affects the decline in annual Forced Expiratory Volume in one second (FEV_1.0_), with declines of −43 mL/year for HTP users and −44 mL/year for cigarette smokers in a study targeting the general population [34]. In contrast, Sakaguchi et al. reported conflicting results, indicating that the level of pulmonary functional parameters of airflow obstruction, such as FEV_1.0_, was significantly higher in the HTP user group than in the cigarette smoker group [35]. Further investigations are still necessary to understand the mechanism behind the harmful effects of HTPs on the worsening of asthma symptoms, but the particle size of HTP aerosols and their distribution primarily affect the lower airways, which is different from cigarettes [29]. Additionally, HTPs have acute harmful effects on small airway function by increasing small airway obstruction and resistance [36]. Furthermore, HTPs may exacerbate airway inflammation and remodeling by altering mitochondrial function in patients with asthma [37]. Thus, HTPs may influence both airway inflammation and airway obstruction, which are the main pathogeneses of asthma, leading to poor asthma control.

Cigarette smoking is associated with poor symptoms and prognosis in asthma control [8–13], which is consistent with our findings. Unlike HTPs, the harmful effects of cigarette smoking on asthma control are well-documented. Cigarette smoking has been associated with various mechanisms that adversely affect individuals with asthma, including narrowed airways, increased wall thickness, altered airway inflammation, and corticosteroid insensitivity [13, 38]. Despite this, higher cigarette smoking rates persist, with reports indicating that most patients with asthma continue to smoke until they experience significant symptoms, such as wheezing, shortness of breath, and discomfort [15]. Additionally, current cigarette smoking is the strongest predictor of HTP use [39]. Our previous study also indicated that cigarette smokers tend to be dual users of cigarettes and HTPs among adults with asthma and COPD [25]. Consequently, it is essential to acknowledge the association between cigarette smoking and poor asthma control.

However, in this study, no significant differences were observed between past cigarette smoking and worsening asthma symptoms, regardless of HTP use among past cigarette smokers. There have been no reports demonstrating an association between HTP use and asthma control among past cigarette smokers. Additionally, some studies have indicated no significant differences in asthma control between former and never-cigarette smokers [9, 40]. This study was a questionnaire-based research, and the group “past cigarettes” is heterogeneous; therefore, further investigations are required to validate our findings.

Increased age is a risk factor for worsening of asthma symptoms [11, 41]. However, the results of this study indicated that younger age was a poor risk factor for worsening of asthma symptoms. Our analysis results were adjusted for confounding factors such as sex, alcohol intake, and BMI, all of which are recognized as risk factors for worsening of asthma control [11, 12, 42, 43]. However, the quality of asthma control is influenced by various personal and/or environmental factors such as treatment contents for asthma, comorbidities, history of asthma exacerbations, mental health status, etc [5, 41]. Similar to our results, Lugogo et al. reported that younger individuals had a significantly higher annualized rate of asthma exacerbation than older adults [43], and this observation may be attributed to the increased prevalence of allergic diseases and nasal polyps in younger adults [4, 43]. Furthermore, the younger age group generally exhibits poorer treatment adherence and a higher prevalence of pet ownership than the older age group [44]. While there may be various opinions in interpreting these results, our findings suggest that younger adults with asthma should be vigilant about worsening of their asthma symptoms.

This study had several limitations. First, it utilized a self-reported internet survey based on questionnaires, which may have led to over- or under-estimation of the diagnoses and disease worsening. However, previous reports have demonstrated a strong correlation between self-reported asthma and clinically confirmed diagnoses [45]. Additionally, the proportion of participants who reported worsening asthma symptoms in this study was approximately comparable with epidemiological data in Japan. Furthermore, responders with discrepancies or inconsistencies in their answers were excluded to improve the accuracy of our study; therefore, we believe that the validity of our internet-based findings has been improved. However, potential biases related to self-reported data and internet-based sampling should still be considered. Second, this study could not evaluate smoking dose, frequency, or duration of cigarettes and HTPs, which may limit the interpretation of dose-response relationships and data on disease onset and duration, asthma phenotype classification, and treatment details for asthma, which may affect the factors involved in asthma control, such as a decline in lung function and treatment efficacy [46–48]. Additionally, asthma is characterized by a hypersensitive airway response to various environmental triggers such as allergens, respiratory viral infection, comorbid allergic diseases, and drugs, and these factors are associated with worsening of asthma symptoms [49]; however, we could not evaluate the influence of these unmeasured confounders. Third, this study was based on cross-sectional data, making it difficult to draw causal inferences. Furthermore, reverse causation cannot be ruled out; for example, patients with poorly controlled asthma may tend to switch from cigarettes to HTPs on the basis that they are a less harmful alternative.

Despite these limitations, to our knowledge, this is the first population-based study to investigate the association between HTP use and worsening asthma symptoms, using data from a nationwide, representative internet-based survey. A major strength of this study is its large scale and diverse sample, including nearly 4,000 self-reported patients with asthma, although the response rate may be relatively low. The JACSIS study was designed to reflect the general Japanese population through an internet panel matched to national demographics, supporting the generalizability of the findings. Future longitudinal studies are required to assess the causal relationships between HTP use and asthma symptoms over time. This would help clarify the temporal dynamics of worsening asthma symptoms in relation to smoking behaviors.

## Conclusions

This study clarified that HTP use was associated with worsening of asthma symptoms in adults, similar to cigarette smoking, although HTPs are often used as substitutes for cigarettes and are mistakenly believed to relieve respiratory symptoms. Therefore, it is important that people understand the harmful effects of HTP use on asthma symptoms. Physicians must also recognize that HTP use negatively affects asthma control, as Japanese physicians may lack sufficient information and knowledge about HTPs [50].

## Abbreviations

BMI: body mass index
COPD: chronic obstructive pulmonary disease
FEV_1.0_: forced expiratory volume in one second
HTPs: heated tobacco products
JACSIS: Japan COVID-19 and Society Internet Survey

## Declarations

### Availability of data and material

The datasets used in the current study are available upon reasonable request.
